# Regeneration and Recyclability of Magnetic Nanomaterials for Multi-Cycle Water Treatment: Toward Circular Adsorption–Desorption Systems

**DOI:** 10.3390/nano16100609

**Published:** 2026-05-16

**Authors:** Mai M. A. Hassan Shanab, Arfa Iqbal, Emre Cevik, Amani M. Alansi, Abdullah M. Aldawsari, Alya M. Alotaibi, Talal F. Qahtan

**Affiliations:** 1 Department of Chemistry, College of Science and Humanities in Al-Kharj, Prince Sattam Bin Abdulaziz University, Al-Kharj 11942, Saudi Arabia; m.hassan@psau.edu.sa (M.M.A.H.S.); abdullah.aldawsari@psau.edu.sa (A.M.A.); aly.alotaibi@psau.edu.sa (A.M.A.); 2 Institute of Environmental Engineering and Research, University of Engineering and Technology, P.O. Box 54890, Lahore 54000, Pakistan; arfa.libra@gmail.com; 3 Bioenergy Research Unit, Department of Biophysics, Institute for Research and Medical Consultations, Imam Abdulrahman Bin Faisal University, P.O. Box 1982, Dammam 31441, Saudi Arabia; ecevik@iau.edu.sa; 4 Department of Chemistry, Faculty of Applied Sciences, Taiz University, Taiz 6803, Yemen; mn.alansi2016@gmail.com; 5 Department of Physics, College of Science and Humanities in AI-Kharj, Prince Sattam Bin Abdulaziz University, Al-Kharj 11942, Saudi Arabia

**Keywords:** magnetic nanomaterials, multi-cycle operation, regeneration techniques, stability, water treatment

## Abstract

Magnetic nanomaterials (MNMs), particularly magnetically recoverable systems with efficient regeneration capability, have emerged as highly efficient nanoadsorbents for water purification owing to their high surface area, tunable surface chemistry, and facile magnetic separation. This review critically analyzes recent advances (2022–2025) in the multi-cycle use of MNMs, with particular emphasis on regeneration strategies. The major syn-thesis approaches and adsorption mechanisms are discussed in relation to their influence on long-term stability. Recent studies demonstrate that many MNMs retain 85–90% of their removal efficiency over 3–6 cycles, although performance degradation due to aggregation, leaching, and surface passivation remains a key challenge. Regeneration techniques, including chemical, solvent-based, and thermal methods, are evaluated in terms of efficiency and feasibility. Moreover, bibliometric analysis reveals the increasing research focus on recyclable nanomaterial design. Overall, this review elucidates the structure–performance–stability relationships governing multi-cycle operation, with a particular focus on reusable and magnetically separable systems and provides insights into the economic feasibility of regenerable MNMs along with future perspectives for sustainable and scalable water treatment applications.

## 1. Introduction

With the accelerated growth in the worldwide population, industrialization, and urbanization, water pollution has intensified as a result of the continuous release of noxious substances into water bodies, which has become an imperative global challenge [1]. A wide range of toxic, non-biodegradable, and persistent contaminants, including heavy metals (HMs), dyes, and pharmaceuticals, are discharged into water owing to anthropogenic activities, which adversely impact human health and the natural ecosystems [2]. To date, several techniques have been explored for water treatment, which encounter various issues in terms of energy requirements, detrimental health effects, high costs, and secondary pollution [3,4].

**Figure 1:**
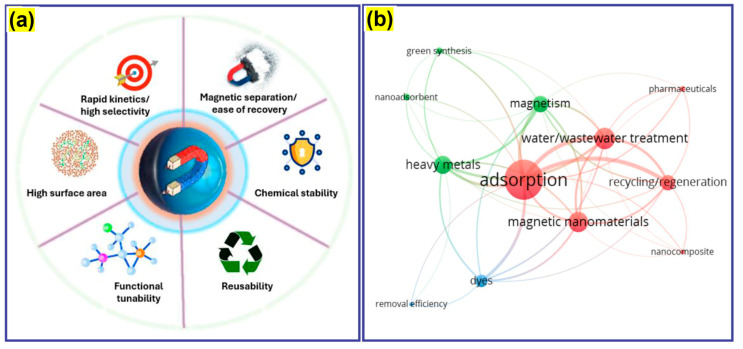
(**a**) Key advantages of MNMs as nanoadsorbents for water treatment and (**b**) network map of keyword cooccurrences in recent investigations on MNMs as nanoadosrbents for water/wastewater treatment, where different colors represent distinct keyword clusters and the connecting lines indicate the relationships and co-occurrence strength among the keywords.

With the advent of nanotechnology during the last decade, a variety of nanomaterials have been studied for water treatment as adsorbents and proven to be very effective due to their high surface area, ability to simultaneously remove multiple pollutants, and remarkable adsorption capacity. From the diverse range of engineered nanomaterials, magnetic nanomaterials (MNMs) are considered quite promising nanoadsorbents for water treatment applications [5] owing to their intrinsic magnetic behavior, as they can be rapidly separated from the system using magnetic fields, making them ideal for developing magnetically recoverable and reusable nanoadsorbents with enhanced regeneration capability for water treatment [6]. The major advantages of MNMs as nanoadsorbents have been presented in Figure 1a.

Despite these functional benefits, the practical application of MNMs heavily relies on their regeneration capabilities and prolonged stability over consecutive adsorption–desorption cycles. The latest investigations conducted on MNMs have focused on iron oxide nanoparticles (Fe_3_O_4_, γ-Fe_2_O_3_), spinel ferrites (NiFe_2_O_4_, CoFe_2_O_4_, MnFe_2_O_4_), and hybrid magnetic composites (polymer-, carbon-, and MOF-based materials). It is important to note that while other magnetic materials such as α-Fe_2_O_3_, nanoscale zero-valent iron (nZVI), and Co/Ni-based oxides have also been explored in adsorption applications, the present review primarily focuses on magnetically recoverable MNMs with demonstrated multi-cycle regeneration performance.

In a recent study, carbon-coated NiFe_2_O_4_ nanoadsorbent showed excellent adsorption performance for the removal of Pb (325.6 mg g^−1^) and Cu (497.7 mg g^−1^) while retaining nearly 79.56% and 82.15% removal efficacy after five regeneration cycles [7]. Similarly, in another study, graphene oxide (GO)-based three-dimensional iron nanoparticles were synthesized via grafting onto melamine (ME) and evaluated against the removal of levofloxacin (LEV), a fluoroquinolone antibiotic. The synthesized nanoadsorbent had an adsorption capacity of 9.72 mg g^−1^ with remarkable regeneration capability (up to 75% after seven adsorption cycles) [8]. These examples signify the potential of MNMs for multi-cycle water treatment.

However, most of the published work primarily emphasizes the adsorption performance of nanoadsorbents only in terms of adsorption capacities while neglecting their regeneration capability, and recyclability. Adsorbents possessing poor regeneration capacity produce secondary pollution and increase operational expenses, whereas MNMs can enhance environmental sustainability owing to their facile magnetic recoverability and excellent potential for regeneration/recyclability. Thus, these features must be considered as key design indicators for commercial-scale sustainable water treatment applications.

Unlike previous reviews that primarily focus on adsorption capacity, this work emphasizes the multi-cycle performance, regeneration efficiency, and long-term stability of MNMs. Therefore, a critical analysis is required to evaluate their reusability and overall stability during consecutive regeneration cycles to ensure sustainable water treatment on a commercial scale. In this regard, the current review covers the latest developments (2022–2025) in recyclable MNMs, concentrating on iron-oxide-based systems (Fe_3_O_4_, γ-Fe_2_O_3_), spinel ferrites (NiFe_2_O_4_, CoFe_2_O_4_, MnFe_2_O_4_), and hybrid magnetic composites (polymer-, carbon-, and MOF-based materials) as nanoadsorbents. This focused scope enables a systematic evaluation of structure–performance–stability relationships in MNMs with efficient magnetic recovery and regeneration behavior.

Iron-oxide-based materials are one of the most important classes of MNMs, followed by spinel ferrites and hybrid magnetic composites. Special attention is given to sustained adsorption performance during multi-cycle water treatment targeting various water pollutants, including HMs, dyes, and pharmaceuticals. Moreover, advances in the design of MNMs, their adsorption mechanisms, and regeneration techniques have also been reviewed while addressing various persistent key challenges over multiple adsorption/desorption cycles like leaching, aggregation, loss of structural integrity, and limited reusability.

To further visualize the emerging investigation trends and thematic relationships within this area, a bibliometric evaluation was performed by developing a network map of keyword cooccurrences from recent publications through VOS viewer software (version 1.6.20) (Figure 1b). The prominent interconnected clusters include adsorption, water/wastewater treatment, recycling/regeneration, magnetism, green synthesis, heavy metals, dyes, and pharmaceuticals. Thus, this network clearly highlights the growing focus on the reusability and sustainable materials design of MNMs for multi-cycle water treatment with promising regeneration capabilities. Finally, future perspectives are outlined, highlighting techno-economic and environmental assessments for practical scale deployment.

## 2. MNMs for Multi-Cycle Adsorptive Removal of Water Pollutants

The role of MNMs in multi-cycle adsorption systems for water treatment depends on two major factors. Firstly, their nanoscale size must allow for efficient superparamagnetic (SPM) behavior to assist in their uniform dispersion and fast separation from water systems after each regeneration cycle [9]. Secondly, the composition of MNMs should be potentially stable to reduce leaching effects during their repeated application, along with suitable surface chemistry, which can improve pollutant binding ability, thereby providing abundant active sorption sites during multiple cycles [10]. In addition to SPM features, the magnetic behavior of MNMs and their hysteresis properties can holistically explain their separation and reusability performance during multi-cycle water treatment. Based on composition and particle size, MNMs depict superparamagnetic, ferromagnetic, or ferrimagnetic behavior, observed through magnetization curves. Different parameters like remanent magnetization (M_r_), saturation magnetization (M_s_), and coercivity (H_c_) are considered important in evaluating magnetic responsiveness [11]. Generally, superparamagnetic materials possess negligible M_r_ and H_c_, whereas M_s_ strongly relies on composition, particle size, crystallinity, and surface modification, exhibiting a typical range between 40 and 90 emu g^−1^ [12]. Their narrow hysteresis loop depicts excellent reusability and redispersion features. However, ferromagnetic or ferrimagnetic materials indicate non-zero M_r_ and H_c_ with broad hysteresis loops, causing particle aggregation and reducing redispersion during reusability studies. Paramagnetic materials, on the other hand, are less efficient due to weak magnetization [13]. Therefore, overall, these parameters play a significant role in determining the recyclability performance of MNMs.

Depending on their compositions and structures, MNMs are mainly classified into three major categories for adsorption-based water treatment systems: iron oxide nanoparticles, spinel ferrites, and hybrid magnetic composites, which are briefly discussed in the following section, with a special focus on their reusability performance during multi-cycle water treatment.

### 2.1. Types of MNMs for Multi-Cycle Water Treatment

Iron oxide nanoparticles are the most widely investigated MNMs throughout the world owing to their distinctive features, including rapid kinetics, high surface area to volume ratio, low toxicity, excellent adsorption capacities, and an additional benefit of strong magnetism, making them suitable for repeated adsorption/desorption cycles. When an external magnetic field is applied, these nanoparticles aggregate rapidly and can be easily separated from the system, enabling facile and cost-effective recovery during multi-cycle treatment. As the magnetic field is removed, they redisperse in the system owing to their SPM behavior, enabling their repeated application during multiple adsorption/desorption cycles. Magnetite (Fe_3_O_4_), and maghemite (γ-Fe_2_O_3_) have excellent SPM features and therefore can either be used directly as nanoadsorbents or employed into nanocomposites [9]. The surface of these nanoparticles can be modified with ligands or chelating agents for the selective removal of water pollutants [10]. However, bare iron oxide nanoparticles can cause aggregation and oxidation, thus affecting their reusability for multi-cycle treatment. These constraints can be overcome by binding them with coupling agents or polymers to enhance their dispersion and stability during repeated adsorption/desorption cycles. Taqui et al. synthesized iron oxide nanoparticles from scrap iron for the removal of Pb ions from aqueous solution. The maximum adsorption capacity achieved was 68.07 mg g^−1^, with excellent reusability (90% removal efficiency after the first two cycles) [14].

In addition to iron oxide nanoparticles, spinel ferrites (MFe_2_O_4_, where M = Ni, Co, and Mn) have caught significant attention from researchers owing to their improved surface and magnetic features, which are essential for their repeated application in multi-cycle adsorption. The partial substitution of Fe^2+^ and Fe^3+^ ions with transition metals alters electron distribution, thereby enhancing redox activity, which is favorable for interactions with HMs and organic pollutants. For example, NiFe_2_O_4_ shows moderate coercivity and magnetization behavior, which is suitable for effective magnetic separation, thereby reducing aggregation issues during adsorption/desorption cycles. Moreover, NiFe_2_O_4_ is resistant to oxidation, highly durable, and flexible for functionalization [15]. CoFe_2_O_4_, on the other hand, has higher coercivity and improved chemical stability compared to Fe_3_O_4_ nanoparticles, which makes it very promising for regeneration and reusability under harsh environments [16]. Another spinel ferrite, MnFe_2_O_4,_ can retain excellent adsorption performance over multiple regeneration cycles due to the existence of lattice defects and additional active sites [17]. The latest investigations indicate that doping approaches and the formation of hybrid nanocomposites can further boost adsorption affinity for water pollutants and active binding sites, along with enhanced magnetic properties for regeneration capabilities. Sheerazi et al. synthesized nicotinamide functionalized CoFe_2_O_4_ nanoadsorbent and observed excellent adsorption capacities, i.e., 177 mg g^−1^ and 101 mg g^−1^ for the removal of two dyes, Bismarck brown (BB) and Celestine blue (CB), from water, sustaining removal efficiency for up to five regeneration cycles, indicating its strong potential for multi-cycle adsorption [18].

Despite the promising adsorptive performance of bare iron oxides and ferrites, they are susceptible to oxidation, cause aggregation, and possess limited structural durability when used for multiple regeneration cycles. These challenges can be overcome by developing hybrid magnetic nanocomposites through the integration of iron nanoparticles with functionalized supports like polymers, carbon, and MOFs. Such hybrid nanocomposites demonstrate exceptional magnetic separation properties together with a high surface area and tunable chemistry, making them multifunctional nanoadsorbents with remarkable selectivity and recyclability for multi-cycle treatment. Polymer-based hybrids like Fe_3_O_4_@alginate, Fe_3_O_4_@polyvinyl alcohol, and Fe_3_O_4_@chitosan offer abundant functional groups (-COOH, -OH, and NH_2_), which assist in binding a wide range of water pollutants. Moreover, their flexible frameworks improve structural robustness and reusability over multiple regeneration cycles. Torasso et al. fabricated unique ultra-small iron oxide nanoparticles within electrospun polymeric nanofibers, which were studied for the removal of As metal ions from water. The adsorption capacity obtained was 3.5 mg g^−1^ with 10 μg L^−1^, whereas the reusability studies found that the removal efficiency of adsorbent remained >80% up to three adsorption/desorption cycles [19]. Carbon-based hybrids, such as Fe_3_O_4_@activated carbon, Fe_3_O_4_@graphene oxide, and Fe_3_O_4_@biochar, couple the magnetic behavior of iron with the intrinsic features of carbon materials involving high surface area, stability, and conductivity, which are crucial for the removal of water pollutants. Furthermore, carbonaceous materials improve resistance to oxidation, thereby limiting the leaching effect during reusability, which enhances their regeneration performance over multiple consecutive cycles. Recently, MOF-based hybrids like Fe_3_O_4_@ZIF-8 and Fe_3_O_4_@MIL-101 have gained the attention of global researchers owing to their diverse structures and outstanding adsorption performance. The tunable functional sites and high porosity of MOFs provide plenty of active adsorption sites for a wide range of organic and inorganic pollutants, whereas the magnetic core assists in fast separation and recyclability, making them excellent candidates for multi-cycle adsorption processes.

### 2.2. Synthesis Approaches for MNMs

**Figure 2:**
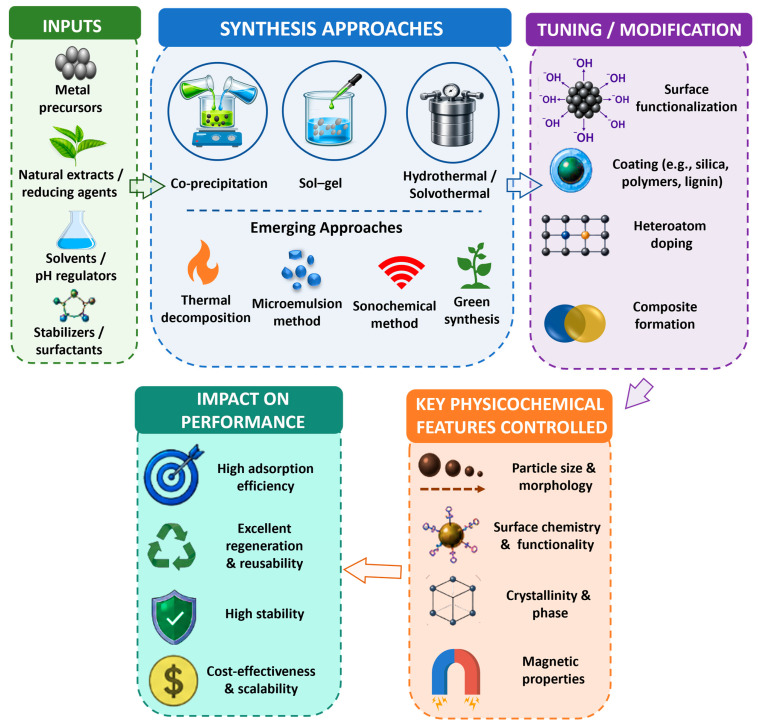
Schematic illustration of the synthesis approaches, post-synthesis modification strategies, and their influence on key physicochemical properties and the adsorption performance of MNMs in multi-cycle water treatment.

Synthesis approaches for MNMs are crucial for determining particle size, surface functionalization, crystallinity, and magnetic features, which significantly impact their adsorption performance during multiple adsorption/desorption cycles. As illustrated in Figure 2, the overall design of MNMs begins with appropriate input materials, including metal precursors, natural extracts or reducing agents, solvents/pH regulators, and stabilizers or surfactants, which collectively influence the synthesis pathway and final material properties. To date, several preparation techniques have been explored for MNMs; however, the common ones include precipitation, sol–gel, and solvothermal/hydrothermal techniques. In addition to these techniques, several other emerging approaches have also been investigated to tailor the physicochemical features of MNMs to improve their regeneration capabilities during multi-cycle water treatment, such as thermal decomposition, the microemulsion method, combustion synthesis, the sonochemical method, and green synthesis. Following synthesis, MNMs are often subjected to various tuning and modification strategies, including surface functionalization, coating (e.g., silica, polymers, or lignin), heteroatom doping, and composite formation, as depicted in Figure 2. These post-synthesis modifications play a critical role in optimizing surface chemistry, enhancing stability, and improving adsorption–desorption behavior during repeated cycles. An overview of synthesis approaches along with their principles, key benefits, and limitations is presented in Table 1.

Recently, Ferenj. et al. employed a green precipitation-based approach to prepare a unique magnetic nanoadsorbent (Fe_3_O_4_/NiO). The leaf extracts of *Hagenia abyssinica* were used as a reducing and stabilizing agent during the synthesis process. The prepared nanoadsorbent had excellent removal efficiency (approximately 97%) for the removal of Pb ions from water. The recovery of the nanoadsorbent after treatment was carried out via an external magnetic field, and removal efficiency remained up to 90% even after the third regeneration cycle [27]. Similarly, Irfan et al. synthesized a silica-coated Cu_0.50_Mg_0.50_Fe_2_O_4_ magnetic nanoadsorbent to study the removal of Pb and Zn ions from water. Firstly, Cu_0.50_Mg_0.50_Fe_2_O_4_ was prepared via a sol–gel method, which was further functionalized with silicate improvement for the stability of the nanoadsorbent. The results demonstrated 97% and 92% removal efficiencies for Pb and Zn, respectively, which stayed >85% after three consecutive cycles, demonstrating outstanding reusability [28]. In another study, a hydrothermal approach was employed for the formation of Fe_3_O_4_–NH_2_, which was further modified with plant-extracted lignin to enhance the adsorption performance of the nanoadsorbent.

The resultant nanoadsorbent had excellent adsorption capacity for the removal of various pharmaceuticals, i.e., 19.2 mg g^−1^, 25.2 mg g^−1^, 33.7 mg g^−1^, and 30 mg g^−1^ for LOM, PEF, DIF, and ENR, respectively, with remarkable reusability up to five regeneration cycles [29]. These examples clearly demonstrate that synthesis approaches, combined with appropriate modification strategies, directly control key physicochemical features, such as particle size, surface functionality, crystallinity, and magnetic properties, which in turn govern adsorption efficiency, regeneration capability, structural stability, and overall cost-effectiveness, as summarized in Figure 2. Thus, all findings suggest that the selection of a suitable synthesis approach is critically important for improving adsorption performance in multi-cycle water treatment.

## 3. Regeneration Strategies for Magnetic Nanomaterial

**Figure 3:**
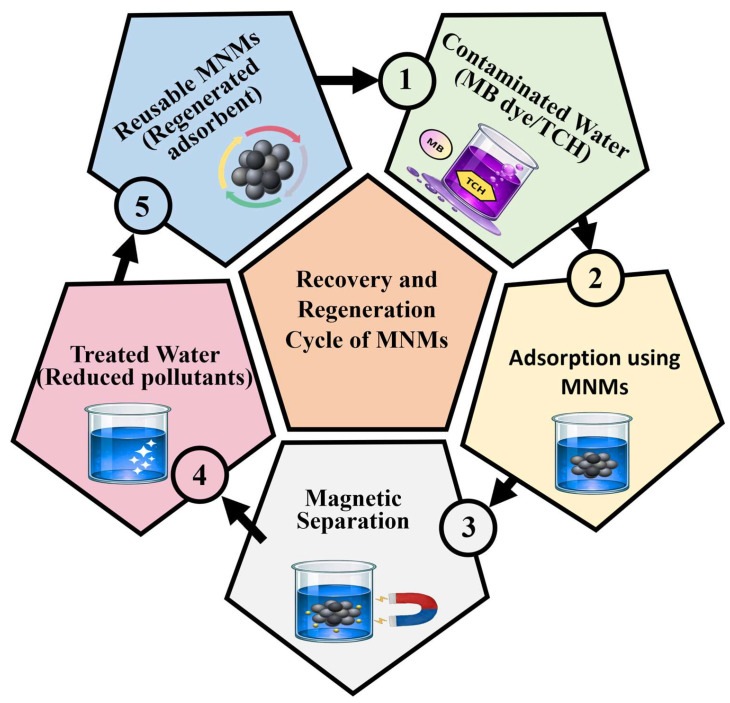
Schematic representation of the adsorption–magnetic separation–regeneration–reuse cycle of MNMs for the removal of pollutants (e.g., MB dye and TCH) in multi-cycle water treatment.

The recyclability of MNMs is considered a crucial factor in multi-cycle water treatment systems. Reusing the exhausted adsorbent not only reduces economic constraints but also provides environmental benefits in terms of sustainable water treatment [30]. As illustrated in Figure 3, the application of MNMs follows a cyclic process that involves pollutant adsorption, magnetic recovery, regeneration, and reuse in successive treatment cycles. It has been observed that MNMs can be employed over multiple adsorption/desorption cycles for the removal of water pollutants; however, their adsorption capacities usually decline after each cycle compared to the new ones, which can be due to morphological changes in nanoadsorbents or limited desorption of pollutants. Therefore, the selection of suitable recovery and regeneration techniques is crucial, which is associated with the type of MNM used, the nature of pollutants, and practical feasibility [31].

### 3.1. Recovery and Regeneration of MNMs

Recovery of MNMs from aqueous solutions is commonly performed via magnetic field assistance and filtration. The most commonly used separation method is magnetic field assistance, which deals with the physical separation of MNMs from water after treatment through the magnetic field. This technique is rapid and efficient and minimizes material loss, enabling effective recovery of nanoadsorbents. As depicted in Figure 3 (Steps 1–2), contaminated water containing pollutants such as dyes (e.g., methylene blue, MB) and antibiotics (e.g., tetracycline, TCH) is first treated using MNMs, where adsorption of pollutants occurs on the nanoadsorbent surface. Moreover, MNMs demonstrate a significant advantage over conventional nanoadsorbents owing to their superparamagnetic properties, which allow for rapid and efficient separation from aqueous systems [32]. Following adsorption (Step 3), the loaded MNMs are separated from the treated solution using an external magnetic field, ensuring efficient recovery of the nanoadsorbent with minimal loss. Filtration may also be applied when nanoadsorbents are highly dispersed in solution; however, magnetic separation remains the preferred method due to its simplicity and effectiveness. These recovery techniques are essential prior to regeneration, enabling subsequent reuse of MNMs [33]. After recovery, regeneration processes are carried out (Step 4), where adsorbed pollutants are desorbed from the surface of MNMs using chemical agents (e.g., acids, bases, or solvents) or thermal treatment, thereby restoring the active adsorption sites. Chemical treatment typically involves washing the spent nanoadsorbent under mild conditions to remove strongly bound contaminants while preserving structural integrity, whereas thermal treatment involves heating to eliminate adsorbed species [34]. Finally, the regenerated MNMs are reused in subsequent treatment cycles (Step 5), demonstrating their recyclability and sustainability in multi-cycle water treatment systems, as highlighted in Figure 3.

Figure 3 illustrates an example of the recovery and regeneration capability of MNMs in the removal of two pollutants, a dye (MB) and an antibiotic (TCH). The nanoadsorbent was recovered by using an external magnetic field, whereas ultrasonic-assisted desorption was carried out in the presence of an eluent solution (0.1 mol L^−1^), demonstrating a feasible strategy for the recovery and regeneration of MNMs [35].

It has been reported that magnetic nanoadsorbents have remarkable regeneration capabilities when treated chemically, maintaining approximately 94% removal efficiency for arsenic (As) after six consecutive cycles [36]. Recently, Hingrajiya et al. prepared a unique Fe_3_O_4_-modified chitosan-based *co*-polymeric magnetic composite hydrogel as a nanoadsorbent for the removal of methylene blue (MB) dye from water. After treatment, the nanoadsorbent was recovered via magnetic field assistance and regenerated through chemical washing. The obtained results not only showed excellent adsorption capacity (860 mg g^−1^) of synthesized nanoadsorbent for the removal of MB but also demonstrated remarkable regeneration capabilities (retaining 92.5% removal efficiency after five adsorption/desorption cycles) [37]. Similarly, Li et al. synthesized core shell Fe_3_O_4_ nanoadsorbent for the removal of Cr ions from water. After adsorption, the nanoadsorbent was physically recovered using a magnet from the treated solution and regenerated via chemical treatment. The adsorption capacity achieved was 13.8 mg g^−1^, retaining 75% after six adsorption/desorption cycles [38]. A summary of the multi-cycle adsorption performance of MNMs for the removal of various types of water pollutants, along with desorbing agents employed for their regeneration, is provided in Table 2.

### 3.2. Factors Affecting Regeneration Efficiency

The regeneration efficiency of MNMs is significantly influenced by various factors. For example, physicochemical features of MNMs like porosity, surface area, particle size, and the presence of functional groups on the adsorbent’s surface are responsible for their stability and desorption effectiveness. Another factor, the nature of pollutants, also plays an important role in determining regeneration efficiency. Strongly adsorbed pollutant ions may need harsh conditions for regeneration compared to weakly bound ions. The stability of an adsorbent is considered another crucial factor, as the loss of active sorption sites and structural variations during repeated adsorption/desorption cycles reduces regeneration capacity. Additionally, prolonged regeneration processes may lead to leaching components from the nanoadsorbent and aggregation. The leaching effect gradually leads to the loss of nanoadsorbent, and aggregation causes blockage of active sorption sites, thereby significantly reducing surface area. Moreover, various operational conditions like temperature, pH, contact time, and type of solvent significantly affect the regeneration capability of the nanoadsorbent. For example, Xu et al. studied the regeneration performance of Fe_3_O_4_@SiO_2_ core–shell magnetic silica nanoparticles modified with organic polymer (MSNPs-CAAQ) for the desorption of chromium (Cr) ions from water for six consecutive adsorption/desorption cycles. Various regeneration agents (HCl, HNO_3_, and Na_2_EDTA) were employed, and maximum removal efficiency (nearly 89%) was achieved with Na_2_EDTA, indicating the effect of the type of desorbing agents on regeneration capabilities [58].

To summarize, the regeneration performance of MNMs primarily depends on the type of adsorbent/pollutants and the operational conditions. Thus, all of these factors must be optimized to achieve sustainable commercial-scale multi-cycle water treatment.

## 4. Multi-Cycle Adsorption Performance

Owing to their adsorptive effectiveness in terms of high surface area and adsorption capacities, easy functionalization, chemical stability, facile separation, and reusability, MNMs have gained immense attention as nanoadsorbents for the removal of a diverse range of water pollutants like HMs, dyes, and pharmaceuticals via multiple mechanisms (electrostatic interactions, hydrogen bonding, ion exchange, π–π interactions, complexation, and hydrophobic interactions [31]) depending on their nature and the type of targeted pollutant like dyes and HMs. It is crucial to completely understand these mechanisms for the efficient design of MNMs exhibiting remarkable adsorption capacities and recovery/regeneration for multi-cycle adsorption process.

Electrostatic interactions occur between oppositely charged nanoadsorbent and pollutant particles. Major environmental parameters that control these interactions include ionic strength, pH, and temperature [59]. Another mechanism, hydrogen bonding, occurs between polar functional groups existing on the nanoadsorbents, like amines, hydroxyl, and carboxyl groups, and the hydrogen acceptor/donner sites of water pollutants, thereby improving adsorption selectivity [60]. Ion exchange mechanisms, on the other hand, refer to the exchange of surface ions of MNMs with the ions of surrounding water contaminants. This exchange ability relies on the density of replaceable ions existing on the surface of MNMs and the overall surface area of MNMs [61]. In π–π interactions, aromatic water contaminants (pharmaceuticals, and dyes) interact and overlap with the aromatic rings of MNM-based nanoadsorbents [62]. Surface complexation/chelation involves the formation of stable coordinate/covalent bonds by water pollutants (like HMs and pharmaceuticals) with the active adsorption sites of MNMs, whereas hydrophobic interactions refer to nonspecific interactions occurring between the non-polar organic pollutants and the hydrophobic surface of nanoadsorbents, avoiding water contact. In many scenarios, multiple adsorption mechanisms occur simultaneously, which have a synergistic impact, thus boosting adsorption performance [63].

The adsorption process is strongly influenced by different parameters, including pH, initial pollutant concentration, adsorbent dosage, temperature, and contact time. Understanding these factors is crucial for optimizing adsorption performance, including adsorption capacity and recyclability [64]. To further gain insight into multi-cycle adsorption behavior regarding quantitative/qualitative aspects, isotherm models, kinetics, and thermodynamic studies are evaluated. By analyzing isotherm models, the adsorption capacity along with the interaction strength between water pollutants and adsorbents can be interpreted. The most commonly employed isotherm models include Langmuir, Freundlich, Sips, Temkin, Flory–Huggins, and Redlich–Peterson models [65]. Kinetic studies provide information about the physical/chemical nature of the sorption phenomenon and assist in calculating the rate constant of the adsorption process. Commonly studied kinetic models are pseudo-first/second order (PFO/PSO) and intra-particle diffusion kinetics [66]. Similarly, thermodynamic studies reveal the endothermic/exothermic nature, spontaneity, and interfacial disorder during sorption [67]. Integration of these findings can lead to the development of sustainable MNMs for commercial-scale multi-cycle water treatment applications.

Table 3 summarizes most recent case studies (2022–2025) of MNMs employed for the adsorptive removal of different types of pollutants, highlighting their synthesis routes, adsorption capacities, mechanisms, and multi-cycle adsorption performance. Although MNMs demonstrate remarkable reusability over multiple adsorption/desorption cycles, their long-term performance is often reduced due to several constraints, including leaching effects, surface passivation, and particle aggregation.

The leaching effect corresponds to the dissolution of the nanoadsorbent or its functional groups into the solution, especially in acidic/alkaline regeneration environments. Weakly/uncoated MNMs are more prone to continual loss of core material due to instability. Besides reducing the overall efficiency of the recyclability process, leaching causes secondary pollution issues by releasing toxic metal ions into the environment [31]. Another major constraint is surface passivation, which refers to the blockage of active binding sites or alterations of the adsorbent’s surface chemistry during multiple regeneration cycles. This happens when the adsorbed water pollutants cannot fully desorb from the adsorbent’s surface during regeneration processes, which limits its reusability over multiple cycles. Moreover, strong desorbing agents can change the chemistry of the adsorbent’s surface, thus reducing the affinity for the water pollutants, which results in poor regeneration capacity [67]. During particle aggregation, MNPs tend to aggregate owing to strong dipole–dipole interactions, leading to the formulation of huge aggregates, which reduces their active binding sites and surface area. Moreover, this aggregation results in reduced magnetic behavior of MNMs, thus causing difficult magnetic separation for recovery after the adsorption process. Furthermore, large aggregates limit the mass transfer of water pollutants to active sorption sites of MNMs, which slows down the reaction kinetics [64].

Recently, Ghosh et al. synthesized reusable magnetic iron oxide nanoparticles (JA-MIONs) via coprecipitation to remove an antibiotic and a dye named tetracycline (TCH) and methylene blue (MB), respectively. The results exhibited remarkable adsorption capacities for both pollutants, i.e., 76.92 mg g^−1^ and 200 mg g^−1^ for MB and TCH, respectively. During regeneration studies, methanol and NaOH were employed as desorbing agents. Over four consecutive adsorption/desorption cycles, the removal effectiveness for MB and TCH decreased from 63.81 to 14.65% and 61.17 to 38.33%, respectively. This reduction corresponds to the strong destructive action of the desorbing agent and loss of some part of the adsorbent during desorption [35]. All of these reusability challenges can be overcome by taking several measures like employing mild desorbing agents to limit the alteration of the adsorbent’s surface and modifying the surface of MNMs with polymers, carbon coatings, surfactants, and functional ligands to improve their stability and overall regeneration capability over multiple adsorption/desorption cycles. Hence, sustaining the recyclability of MNMs heavily relies on appropriate regeneration parameters to maintain a balance between the adsorbent’s stability and desorption efficiency.

**Figure 4:**
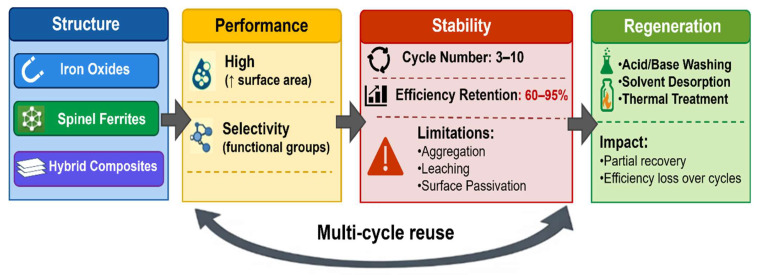
Structure–performance–stability map of magnetic nanomaterials for multi-cycle water treatment, highlighting regeneration pathways and efficiency retention. The upward arrow (↑) indicates increased surface area.

To provide a comprehensive understanding of the relationship between material structure, adsorption performance, stability, and regeneration behavior, a structure–performance–stability mapping approach is presented in Figure 4.

As illustrated in Figure 4, the long-term performance of magnetic nanomaterials is strongly governed by their structural design and regeneration strategy. Hybrid magnetic composites typically demonstrate superior stability and recyclability compared to bare iron oxides due to reduced aggregation, leaching, and surface passivation.

**Figure 5:**
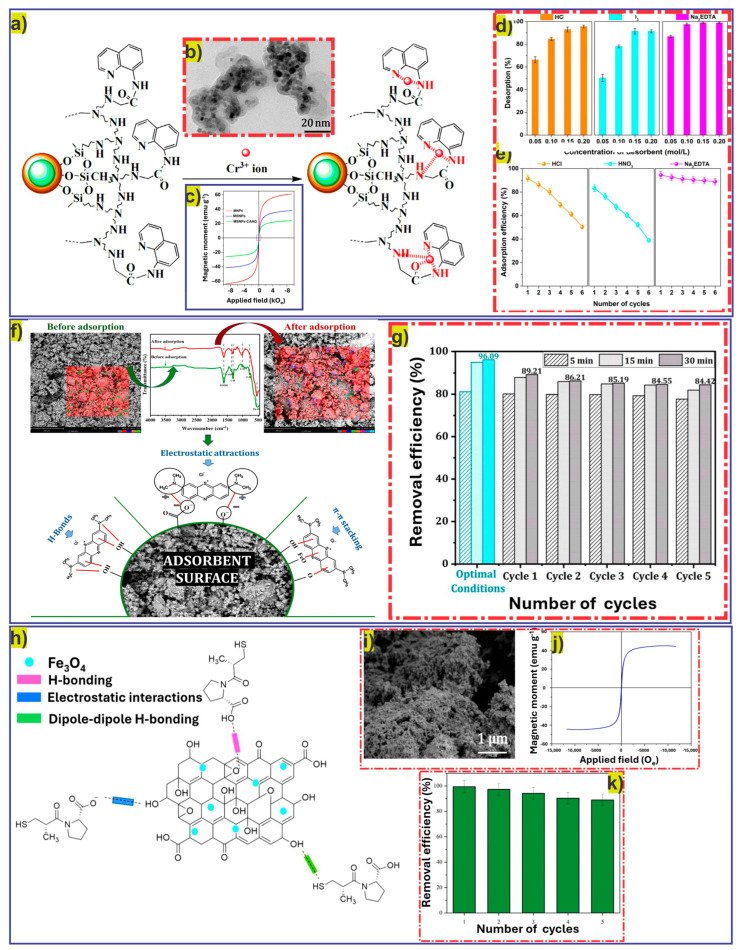
(**a**) Possible interactions between Cr ions and MSNPs–CAAQ, (**b**) TEM analysis of MSNPs–CAAQ, (**c**) magnetization curves for MNPs, MSNPs, and MSNPs–CAAQ, (**d**) effects of various desorbing agents (HCl, HNO_3_, and Na_2_EDTA) on the desorption of Cr ions from the surface of MSNPs–CAAQ, (**e**) reusability test (over six regeneration cycles) for MSNPs–CAAQ using different desorbing agents [58], (**f**) adsorption mechanisms for the removal of MB molecules using cactus/iron oxide nanoadsorbent, (**g**) reusability test (over five regeneration cycles) for cactus/iron oxide nanoadsorbent [68], (**h**) adsorption mechanism for CPT onto GO.Fe_3_O_4_ nanoadsorbent, (**i**) SEM analysis and (**j**) magnetization curve and (**k**) reusability test (over five regeneration cycles) for GO.Fe_3_O_4_ nanoadsorbent [50].

Xu et al. synthesized polymer-modified superparamagnetic magnetic-silica-based nanoadsorbent (MSNPs–CAAQ) and observed excellent removal of Cr ions from aqueous solution with high adsorption capacity (293.3 mg g^−1^). Figure 5a shows the selective coordination of Cr ions with nitrogen and carbonyl oxygen atoms on the surface of the nanoadsorbent. TEM analysis (Figure 5b) of MSNPs–CAAQ confirms the amorphous coating of 8-hloroacetyl–aminoquinoline (CAAQ) around the NPs, which was employed for trapping Cr ions. The magnetic behavior (Figure 5c) of MSNPs–CAAQ exhibits magnetic saturation of 24 emu g^−1^, which is lower than the values of superparamagnetic magnetic nanoparticles (MNPs) and core–shell magnetic silica nanoparticles (MSNPs), which were 61 emu g^−1^ and 38.1 emu g^−1^, respectively. The low value of magnetic saturation for MSNPs–CAAQ was subjected to the existence of non-magnetic layers around the nanoadsorbent. Figure 5d presents the effects of various desorbing agents (HCl, HNO_3_, and Na_2_EDTA) with varying concentrations (0.05–0.20 mol L^−1^) on the desorption of Cr ions from the nanoadsorbent (MSNPs–CAAQ). Desorption efficiency improved with the increasing concentration of desorbing agents. Overall, Na_2_EDTA demonstrated superior performance (98–99% desorption), followed by HCl and HNO_3_ (at high concentrations). Furthermore, the adsorption/desorption performance of MSNPs–CAAQ for Cr ions was also studied for six consecutive regeneration cycles using these desorbing agents (Figure 5e). The nanoadsorbent retained its stability and exhibited excellent adsorption efficiency over six cycles (>85%) with Na_2_EDTA (0.1 mol L^−1^) compared to HCl and HNO_3_. These findings indicate the remarkable potential of MSNPs–CAAQ nanoadsorbent in the removal of Cr ions along with its excellent regeneration capability over repeated cycles [58].

In another study, Boumezough et al. prepared a cost-effective nanoadsorbent (cactus/iron oxide) based on a type of cactus (*Opuntia ficus indica*) modified with MNPs and evaluated its adsorption performance for the removal of MB dye molecules from water. Figure 5f shows that the adsorption phenomenon primarily occurred via electrostatic interactions as the cationic dye molecules attracted the negatively charged -OH and -COOH functional groups existing on the surface of the nanoadsorbent (cactus/iron oxide), confirmed by the modifications observed during FTIR analysis (before and after adsorption). In addition, hydrogen bonding and π-π stacking also contributed to the adsorption mechanism and further enhanced adsorption efficiency. Figure 5g shows that the synthesized nanoadsorbent also demonstrated excellent regeneration efficiency over five successive adsorption/desorption cycles, as a mild decline was observed from 96.09% (first cycle) to 84.42% (fifth cycle) [68].

The comparative analysis in Table 3 shows that adsorption conditions vary considerably across different MNM-based adsorption systems. Among different studies, the pH values ranged between 2 and 10 on average depending on the type of water pollutant removed and the surface of the nanoadsorbent. Similarly, the majority of adsorption studies have been performed under ambient temperature conditions between 25 °C and 30 °C, although some nanoadsorbents showed excellent adsorption at elevated temperatures. Moreover, functionalized MNMs exhibited more stability and excellent regeneration capability over repeated cycles compared to pristine MNMs, which deteriorated faster during repeated usage. These findings clearly indicate that the stability and regeneration capacity of MNMs depend on their composition and materials rather than being uniform across all adsorption systems.

Similarly, Oliveira et al. [50] prepared another magnetic GO-based nanoadsorbent (GO.Fe_3_O_4_) for the removal of a hydrophilic drug, captopril (CPT), from water. The maximum adsorption capacity achieved was 100.41 mg g^−1^, whereas the sorption phenomenon was found to be spontaneous/exothermic and best suited to a Sips isotherm and PSO model. Figure 5h shows the various mechanisms involved during the sorption of CPT onto MGO, which include electrostatic interactions, dipole–dipole interactions, and Yoshida hydrogen bonding. The SEM analysis in Figure 5i confirms the coverage of the GO surface with magnetite Fe_3_O_4_ nanoparticles, whereas the magnetization profile in Figure 5j shows the ferromagnetic behavior of GO.Fe_3_O_4_ nanoadsorbent with a value of 45 emu g^−1^. Desorption of nanoadsorbent was carried out to evaluate its regeneration capability by using an alkali desorbing agent, NaOH (0.25 mol L^−1^). The obtained pure nanoadsorbent was further employed for five consecutive adsorption/desorption cycles (Figure 5k), and the results indicate the outstanding recyclability of the nanoadsorbent (nearly 90% after five cycles).

**Table 3:** Comparative summary of MNMs recently employed (2022–2025) for the removal of various types of water pollutants, including synthesis routes, adsorption capacities, mechanisms, and multi-cycle adsorption performance.

Type of MNM	Synthesis Routes	Pollutant Removed	Ads. Capacity (mg g^−1^)	Ads. Conditions (Ads. Dose/Initial Adsorbate Concentration/pH/Contact Time/Temp)	Multi-Cycle (C) Performance (Removal per Cycle %)	Adsorption Mechanism	Key Observations	Ref.
Fe_3_O_4_@PGP-NPs	Green synthesis	Dye (AG 25)	213	2.2 g L^−1^/200 ppm/6.2/88 min/25 °C	C-1: 97.57%, C-2: 92.23%, C-3: 85.79%, C-4: 85.79%, C-5: 62.23%	Electrostatic interactions and hydrogen bonding via surface functional groups (–OH, C=O/C–O, and Fe–O)	Gradual decrease in removal efficiency	[39]
Fe_2_O_3_ nanoparticles	Green synthesis	HM (Pb, Cd)	2.75, 3.48	0.05 g/2.5 ppm/10/30 min/25 °C	Pb: C-1: 96.8%, C-2: 78.3%, C-3: 69.4%, C-4: 52.1% Cd: C-1: 92%, C-2: 75%, C-3: 60%, C-4: 41.3%	Chemisorption via surface complexation and electrostatic interactions with Fe–OH active sites	High initial metal removal, which declined over successive regeneration cycles owing to strong chemisorption-driven binding between metals and the adsorbent	[41]
IONPs	Green synthesis	HM (Pb, Cd)	118.76, 105.93	1 g/85.5 ppm/6/90 min/25 °C (Pb), 1 g/120 ppm/5/120 min/25 °C (Cd)	Pb: C-1: 95%, C-2: 94%, C-3: 93%, C-4: 91%, C-5: 88% Cd: C-1: 85%, C-2: 84%, C-3: 84%, C-4: 82%, C-5: 80%	Chemisorption via surface complexation and electron exchange with Fe-based active sites (monolayer adsorption)	High adsorption capacity with minimal loss in removal efficiency during regeneration cycles, signifying excellent reusability	[42]
O-Fe_3_O_4_	Green synthesis	Pb	90.10	0.2 g L^−1^/25 ppm/5.5/90 min/55 °C	C-1: 97%, C-2: 94%, C-3: 90%, C-4: 87%, C-5: 83%	Electrostatic interactions and chemisorption via surface functional groups (OH, –COOH) on Fe_3_O_4_ nanoparticles	Gradual decrease in removal efficiency during multiple cycles with overall good regeneration capability	[43]
Fe_3_O_4_@SiO_2_-(TCT)_2_-(NH_2_)_2_ nanoparticles	Precipitation–sol–gel-surface functionalization	HM (Cu, Cd)	99.7, 115.7	16 mg/0.45 mmol L^−1^/7/18 min/25 °C (Cu), 16 mg/0.3 mmol L^−1^/7/20 min/25 °C (Cd)	Cu C-1: 95%, C-2: 94%, C-3: 94%, C-4: 93%, C-5: 93%, C-6: 91%, C-7: 90%, C-8: 88% Cd C-1: 91%, C-2: 91%, C-3: 90%, C-4: 89%, C-5: 89%, C-6: 87%, C-7: 86%, C-8: 84%	Chemisorption via coordination/chelation with NH_2_-containing dendrimer and surface Si–OH groups, supported by electrostatic interactions	Efficient reusability during repeated regeneration cycles owing to functionalization of the adsorbent’s surface	[45]
Fe_2_O_3_ nanoparticles	Green synthesis	Pharmaceutical (TC, DF)	56.68, 62.36	---	TC C-1: 87%, C-2: 82%, C-3: 77%, C-4: 71%, C-5: 66% DF C-1: 90%, C-2: 84%, C-3: 76%, C-4: 70%, C-5: 65%	Electrostatic interactions, hydrogen bonding, π–π interactions, and surface complexation between antibiotic molecules and Fe_2_O_3_ surface functional groups	Moderate regeneration capability owing to gradual decline in removal efficiency during each cycle	[47]
Mn-doped SPIONPs	Green synthesis	HM (Pb)	29.56	100 g L^−1^/500 ppm/5.6/60 min/25 °C	C-1: 87.26%, C-2: 83.1%, C-3: 81.14%, C-4: 76.76%	Chemisorption involving surface interactions between Pb^2+^ ions and active sites	Steady decrease in regeneration performance throughout four cycles	[48]
Fe_3_O_4_@C	Co-precipitation–ultrasonic-assisted surfactant functionalization	Dye (MB)	445	0.01 g/500 ppm/10/120 min/60 °C	C-1: 54.7%, C-2: 54.5%, C-3: 54.5%, C-4: 54%, C-5: 48.4%	Electrostatic interactions and van der Waals (dispersion) forces between MB molecules and functionalized Fe_3_O_4_ surface	Excellent adsorption capacity and moderate regeneration performance with minor decline in removal efficiency over repeated cycles	[49]
Spirulina-assisted mesoporous iron oxide nanoparticles (MIONPs)	Co-precipitation–green synthesis	HM (Cr)	92.85	0.21 g/50 ppm/2/100 min/70 °C	C-1: 78%, C-2: 62%	Electrostatic adsorption of Cr(VI) oxyanions + surface complexation; partial reduction to Cr(III) and immobilization	Considerable decline in just two regeneration cycles exhibiting limited multi-cycle adsorption	[69]
Mesoporous Fe_3_O_4_/PRD hybrid microspheres	Solvothermal–etching–in situ polymerization	HM (Cr)	199	0.21 g/50 ppm/2/100 min/25 °C	C-1: 100%, C-2: 97%, C-3: 96%, C-4: 94%, C-5: 92%, C-6: 91%, C-7: 90%, C-8: 90%	Coordination/complexation with polymer functional groups, electrostatic attraction, pore diffusion	High adsorption capacity with stability in regeneration performance over multiple cycles	[70]
MGO	Oxidation–exfoliation– co-precipitation	Pharmaceutical (CPT)	100.41	50 mg/50 ppm/3/60 min/25 °C	C-1: 99%, C-2: 98%, C-3: 97.5%, C-4: 96%, C-5: 95%	Electrostatic interactions and hydrogen bonding between CPT molecules and oxygenated functional groups on GO–Fe_3_O_4_ nanocomposite surface	Excellent reusability observed over multiple regeneration cycles	[50]
Fe-based MOF (MIL-100)	Reduction	HM (As)	137.7	0.4 g L^−1^/20 ppm/7/60 min/25 °C	C-1: 99%, C-2: 98.5%, C-3: 98%, C-4: 97.5%, C-5: 97.3%	Inner-sphere monodentate Fe–O–As complexation at unsaturated Fe sites	Stable regeneration performance with minimal loss in removal efficiency	[51]
Fe_3_O_4_/NiO	Green synthesis	HM (Pb)	751.88	0.09 g/100 ppm/8/80 min/25 °C	C-1: 97.24%, C-2: 94.12%, C-3: 89.56%	Electrostatic attraction and surface complexation with deprotonated –OH/–COOH groups	Remarkably high adsorption capacity with moderate regeneration capability due to gradual decrease in removal efficiency during multiple cycles	[27]
USMNs	Green hydrothermal synthesis	HM (Pb)	315.43	10 mg/10 ppm/9/60 min/25 °C	C-1: 99.69%, C-2: 98.37%, C-3: 97.13%, C-4: 95.7%, C-5: 91.8%	Electrostatic interactions, surface complexation, and ion exchange with –OH groups	High adsorption capacity and stable regeneration performance over repeated cycles with minimal loss in Pb removal	[52]
Fe_3_O_4_@SiO_2_-NH-nNG-SPTZ	Surface functionalization	HM (Hg)	67.35	0.1 g/100 ppm/7/15 min/25 °C	C-1: 100%, C-2: 96%, C-3: 89%, C-4: 78%, C-5: 55%	Heterogeneous multilayer adsorption via surface complexation/chelation with N/O/S groups and electrostatic attraction	Reduced multi-cycle performance due to significant decline in removal efficiency over repeated cycles	[53]
F/α/C nanocomposite	Co-precipitation	HM (Pb)	731	5 mg/50 ppm/9/20 min/52 °C	C-1: 99.8%, C-2: 99.8%, C-3: 99%, C-4: 97%	Electrostatic attraction + surface complexation (–OH, –COOH) + ion exchange + pore diffusion + pH-dependent precipitation of Pb(OH)_2_ on porous Fe–carbon composite	Excellent adsorption capacity and multi-cycle performance during regeneration, maintaining overall stability in removal efficiency	[54]
Alg/Clin/Fe_3_O_4_	Co-precipitation–ion gelation	Dye (CV)	16.52	5 mg/50 ppm/9/20 min/52 °C	C-1: 92%, C-2: 90%, C-3: 88%, C-4: 85%, C-5: 83%	Electrostatic attraction (due to OH/COO^−^ groups), ion pairing with alginate carboxylates, and pore diffusion in porous Clin/Fe_3_O_4_-based composites	Moderate regeneration capability due to gradual decline in removal performance over multiple adsorption/desorption cycles	[55]
GO/Ni-Fe	Co-precipitation	Pharmaceutical (DXC)	13.02	0.1 g L^−1^/0.2 ppm/5/20 min/25 °C	C-1: 90%, C-2: 90%, C-3: 90%, C-4: 89.5%, C-5: 89%, C-6: 88.5%, C-7: 88%, C-8: 88%, C-9: 88.5, C-10: 87%	Electrostatic interactions, π–π interactions, hydrogen bonding	Excellent reusability retained across multiple regeneration cycles with minimal decline in removal efficiency	[71]
CuMgFe_2_O_4_	Sol–gel	HM (Pb, Zn)	56.68, 51.84	0.25 g/7/36 h/25 °C	Pb C-1: 97%, C-2: 94%, C-3: 88%, Zn C-1: 92%, C-2: 90%, C-3: 85%,	Complexation, electrostatic interactions, ion exchange	Moderate regeneration performance with steady decline in removal efficiencies for both metals over repeated cycles	[28]
Fe_2_O_3_/AC nanocomposite	Green synthesis	Dye (CR)	9.21	0.75 g L^−1^/100 ppm/3/30 min/20 °C	C-1: 98%, C-2: 95%, C-3: 92%,	Hydrogen bonding, electrostatic attraction (–COO^−^, C=O), and π–π stacking		[56]
NiO–Fe_3_O_4_ NPs	Co-precipitation	HM (Pb, Hg)	182.79, 179.92	100 mg/30 ppm/5/30 min/25 °C (Pb), 100 mg/30 ppm/6/30 min/25 °C (Hg)	Pb C-1: 98%, C-2: 97%, C-3: 96%, C-4: 95% Hg C-1: 99%, C-2: 98%, C-3: 97%, C-4: 96%	Electrostatic attraction on deprotonated Fe–OH/Ni–OH sites and chemisorption (inner-sphere complexation)	Steady decline in removal efficiency over repeated cycles, indicating moderate reusability performance	[57]
3D/Fe/GO/ME/DCS	In situ precipitation–grafting	Pharmaceutical (LEV)	9.72	1 g L^−1^/5 ppm/7/30 min/20 °C	C-1: 100%, C-2: 95%, C-3: 92%, C-4: 90%, C-5: 88%, C-6: 86%, C-7: 84%	Monolayer physisorption on a homogeneous surface governed by weak interactions (van der Waals/hydrogen bonding)	Gradual decrease in cyclic regeneration performance	[8]
Fe_3_O_4_@DAL	Hydrothermal grafting	Pharmaceutical (PEF, LOM, ENR, DIF)	25.2, 19.2, 30, 33.7	2.5 g L^−1^/50 ppm/10/1400 min/25 °C	PEF C-1: 95%, C-2: 92%, C-3: 88%, C-4: 85%, C-5: 82% LOM C-1: 97%, C-2: 94%, C-3: 90%, C-4: 87%, C-5: 84% ENR C-1: 98%, C-2: 95%, C-3: 92%, C-4: 89%, C-5: 86% DIF C-1: 99%, C-2: 97%, C-3: 95%, C-4: 93%, C-5: 91%	Electrostatic attraction between –COO^−^/–O^−^ and =N^+^ groups and hydrophobic interactions between FQs and aromatic DAL domains	Steady decline in removal efficiencies for all pharmaceuticals over multiple regeneration cycles, indicating good reusability	[29]

## 5. Economic Feasibility/Challenges in Multi-Cycle Water Treatment

**Figure 6:**
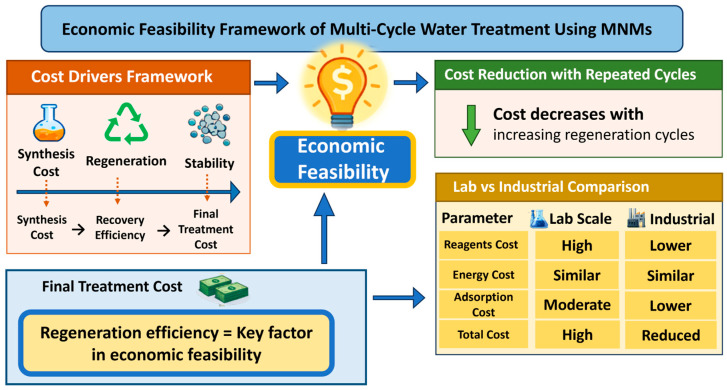
Conceptual framework illustrating the key factors governing the economic feasibility of multi-cycle water treatment using MNMs, including cost drivers, regeneration efficiency, and scale-up effects.

Although MNMs demonstrate remarkable potential for multi-cycle adsorption in the removal of various water pollutants, their economic feasibility and large-scale applicability remain challenging due to several factors, including synthesis cost, regeneration efficiency, recovery effectiveness, and material stability over repeated cycles. The economic performance of MNMs is governed by the interplay between these parameters, which col-electively determine the overall treatment cost. As illustrated in Figure 6, this relationship can be understood through a cost drivers framework that integrates synthesis cost, regeneration behavior, and structural stability. One of the key considerations in evaluating economic feasibility is the balance between the initial synthesis cost and the cost of treatment per unit volume of water. Conventional synthesis methods are often associated with high energy consumption and the use of costly precursors. Therefore, green synthesis strategies are increasingly explored to reduce costs by utilizing low-cost raw materials, minimizing solvent usage, and lowering energy demand. However, challenges related to scalability and reproducibility still limit their widespread industrial application [72]. Beyond synthesis, the economic feasibility of MNMs is strongly influenced by their reusability over multiple regeneration cycles. Improved regeneration efficiency significantly reduces the cost per treatment cycle, as repeated reuse minimizes the need for continuous material replacement. In this context, treatment cost typically decreases with increasing number of regeneration cycles, highlighting the importance of long-term stability and effective regeneration strategies.

Furthermore, the overall cost is influenced by operational conditions and recovery efficiency. Inefficient recovery processes and gradual material loss during repeated cycles can negatively affect cost-effectiveness. In contrast, efficient magnetic separation provides a practical advantage by enabling rapid recovery with minimal energy input. In addition, economic performance varies between laboratory-scale and industrial-scale operations. At larger scales, reagent-related costs are generally reduced due to bulk production and process optimization, while energy costs tend to remain relatively consistent. The combined effect of scale-up and enhanced reusability leads to a progressive reduction in total treatment cost [73,74]. Overall, the framework presented in Figure 6 emphasizes that optimizing regeneration efficiency, ensuring material stability, and improving scalability are essential factors for achieving cost-effective and sustainable multi-cycle water treatment using MNMs.

Table 4 provides insight into the comparative economic evaluation of MNM-based nanoadsorbents in the removal of various HMs, dyes, and pharmaceuticals. It indicates varying adsorption capacities and estimated costs of nanoadsorbents along with the cost of the adsorption process across different MNMs employed for the removal of different water pollutants. Moreover, magnetic recovery adds a significant benefit, as it facilitates the separation of magnetic components via the magnetic field rather than employing energy-intensive techniques like filtration and centrifugation. Consequently, these MNM-based nanoadsorbents lead to low operational costs and sustainable water treatment systems.

## 6. Environmental Implications and Future Research Directions

### 6.1. Structural Stability of MNMs

MNMs offer substantial environmental advantages in multi-cycle water treatment, but several potential risks related to their synthesis, application, and disposal stage must be carefully addressed before commercial-scale application. Although MNMs demonstrate exceptional adsorptive performance for the removal of a broad variety of water contaminants under laboratory-scale investigations, they still face several challenges, such as instability under variable environmental parameters, resulting in the discharge of toxic metal ions into water systems, which limit large-scale multi-cycle water purification applications. Moreover, surface functionalization and different modifications of nanoadsorbents may deteriorate when employed during multiple regeneration cycles, causing ecotoxicological effects [31]. Leaching/dissolution of MNMs during repeated regeneration cycles can potentially affect their reusability. This is generally analyzed via Inductively Coupled Plasma (ICP) analysis for the quantification of metal ions. Studies indicate that MNMs have low dissolution losses over continuous regeneration cycles, typically within a minor fraction of the total nanoadsorbent. However, such quantitative assessments are not widely available. Even minimal losses can adversely affect adsorption performance over repeated usage. Moreover, magnetic hysteresis loop measurements can further provide deep insights into the magnetic behavior of nanoadsorbents during each regeneration cycle. Therefore, evaluating the dissolution behavior as well as the magnetic behavior of MNMs is very important to assess their practical applicability in multi-cycle water treatment systems.

### 6.2. Static vs. Dynamic Adsorption Systems

Moreover, most of the literature related to MNMs is associated with static batch adsorption experiments and thus unable to completely demonstrate practical water treatment systems, which commonly employ dynamic fixed-bed/column-based adsorption (with crucial factors affecting process performance including mass transfer and flow rate). Thus, it is crucial to expand future investigations towards dynamic adsorption studies to better understand the practical implications and stability of MNMs in field-scale water treatment systems.

### 6.3. Gap Between Lab-Scale Studies and Real-World Water Treatment Systems

Investigations conducted under lab-scale controlled conditions cannot entirely represent real-world water treatment systems. The latest case studies between 2022 and 2025 mainly focus on laboratory-scale applications, overlooking the complexities involved in real-world applications, which include varying ionic strengths and co-existing ions. This constraint impedes the complete understanding of the behavior of MNMs, their stability, and their reusability over successive regeneration cycles during real-world multi-cycle water treatment. As a consequence, the removal efficiencies of MNMs reported under lab-scale conditions are overstated in comparison to those achieved in commercial-scale water treatment systems, thus limiting their practical feasibility. Therefore, future research must focus on realistic environmental conditions to better understand the practical application of MNMs.

### 6.4. Ecotoxicological Impacts and Biosorption Feasibility

MNMs induce varying impacts on biological systems, which mainly depend on their type, composition, surface functionalization, and exposure concentration. Surface-modified MNMs usually have low toxicity impacts compared to bare, uncoated nanoparticles, which can result in the release of metal ions into the water and oxidative stress within microbes [83,84]. Furthermore, MNMs can be incorporated with biosorbents for hybrid water treatment approaches [85]. However, careful optimization is necessary to prevent microbial inhibition. Moreover, surface modification of MNMs can help in reducing the toxicity, thereby enabling synergistic biosorption performance.

### 6.5. Life Cycle Assessment (LCA)

Life cycle assessment (LCA) in this regard can provide deep insights into the economic and environmental impacts of synthesis and application, as well as end-of-life management. Various critical environmental parameters, such as water/carbon footprints, ecotoxicological effects, energy consumption, and resource depletion, must be assessed during LCA evaluation. Considering recent years (2022–2025), very few studies report LCAs, while the majority of the investigations only focus on laboratory-scale synthesis and applications, thus revealing a huge gap between batch-scale and commercial-scale deployment. Through LCA, scientists can ensure sustainability and minimize overall process costs by using green precursors in synthesis routes and adopting energy-effective processing [86].

### 6.6. Circular Water Treatment

Another important aspect is related to the integration of MNMs into circular water purification systems, a transformative strategy for sustainable water treatment, promoting a closed-loop framework by minimizing waste generation and maximizing recovery of nanoadsorbents. In multi-cycle water treatment, these MNMs are magnetically separated and reused during multiple adsorption/desorption cycles, resulting in valorization of treated water and the collected concentrate containing valuable HMs and other minerals, thus promoting reusability and less waste generation. This recyclability of MNMs fully aligns with the principles of the circular economy. In addition, hybrid treatment systems consisting of a combination of MNMs with other advanced purification technologies like photocatalytic degradation, membrane filtration, and advanced oxidation processes can further improve the effectiveness of multi-cycle water treatment. For instance, multifunctional water purification systems can be introduced by combining MNMs with membrane filtration, as MNMs can be helpful in adsorbing water pollutants whereas the membrane enables physical separation of pollutants. This synergistic impact not only enhances pollutant removal efficiency but also promotes facile magnetic separation for adsorbent recovery, which supports multi-cycle adsorption [87,88].

### 6.7. Proposed Strategy for Real-World Applications

A multistage strategy is proposed to facilitate the transition of MNM-based nanoadsorbents from laboratory-scale studies to industrial-scale water treatment applications. This framework includes the following key steps:

- Lab-scale validation under realistic conditions using real water matrices to evaluate adsorption performance in the presence of competing ions and complex compositions.
- Hybrid integration with existing treatment systems, where MNMs are incorporated as supplementary materials alongside conventional technologies to enhance removal efficiency without major system modifications.
- Pilot-scale evaluation under dynamic (continuous flow) conditions, enabling assessment of the mass transfer limitations, hydraulic performance, reusability, and structural stability of MNMs.
- Techno-economic and environmental feasibility analysis, including cost assessment, regeneration efficiency, and life cycle considerations.
- Gradual implementation in selective treatment scenarios where MNMs demonstrate superior performance compared to conventional adsorbents.

This stepwise approach provides a practical pathway for the progressive adoption of MNMs, ensuring improved performance while minimizing disruption to existing water treatment infrastructure.

### 6.8. Future Outlook

Future investigations must focus on the formulation of multifunctional MNMs exhibiting long-term performance with effective regeneration capabilities in multi-cycle water treatment systems. Investigations must explore green synthesis techniques, easy recovery methods to avoid nanoparticle leaching, and long-term adsorption performance over repeated cycles under practical conditions. Transformation of MNMs from batch scale to real-life multi-cycle water treatment applications requires optimization of ecofriendly and low-cost synthesis strategies along with the development of closed-loop water reuse systems. To further improve the reliability of reusability studies, future research should focus on systematic characterization frameworks involving the quantification of metal leaching (through ICP), the evaluation of colloidal stability through zeta potential and hydrodynamic diameter before/after each regeneration cycle, and morphological assessment (TEM/SEM images) of fresh and regenerated nanoadsorbents to observe surface modifications.

## 7. Conclusions

This mini review indicates that long-term application of MNMs relies on their structural stability and regeneration capacity over multiple cycles. Although various MNM-based nanoadsorbents exhibit excellent reusability, retaining 85–90% efficiency even after an average of three to six regeneration cycles, several constraints, such as loss of adsorbent and gradual decline in adsorption performance over repeated cycles, remain serious concerns. Chemical regeneration is considered the most common approach for recovering MNM-based nanoadsorbents. The bibliometric network map further illustrates the growing literature on the sustainable design of MNMs and their reusability. Surface functionalization, suitable synthesis routes, and wise selection of desorbing agents can significantly enhance the multi-cycle adsorption process. Moreover, the economic feasibility of MNMs heavily relies on their robustness and efficient reusability, which can decrease overall water treatment costs. Future directions must focus on the scalable design of MNMs along with LCA and techno-economic evaluations for industrial-scale applications. Overall, MNMs have proven to be promising nanoadsorbents for sustainable multi-cycle water remediation applications.

## Figures and Tables

**Table 1:** Synthesis strategies employed for the preparation of different types of MNMs, including their principles, key benefits, and limitations.

Synthesis Approach	Principle	Key Benefits	Limitations	Ref.
Chemical precipitation	Alkaline precipitation of metallic salts	Scalable, facile, economically viable	Particle aggregation, poor control over morphology	[19]
Sol–gel	Involves hydrolysis/condensation of metallic precursors transforming into gel	Produces pure and uniform MNMs, requires low temperature and energy	Time-consuming, moisture sensitivity	[20]
Solvothermal/ hydrothermal	Elevated temperature/pressure reactions in autoclave	Simple operation, controlled morphology	Expensive autoclave, poor yield	[21]
Thermal decomposition	Decomposition of organometallic precursors in organic solvents at high boiling points	Produces monodisperse MNMs, controlled morphology	High cost, requires organic solvents	[22]
Microemulsion	Small water droplets stabilized by surfacing agents act as nanoreactors in which reactions take place	Controlled particle sizes, stable, high purity	Huge volumes of solvents required, reduced scalability, aggregation problems	[23]
Sonochemical synthesis	Strong ultrasonic irradiations to induce chemical reactions	Fast reaction, improves nucleation	Energy-intensive, reduced scalability	[24]
Combustion synthesis	Involves intense exothermic reactions	Economically viable, fast reaction, scalable for bulk synthesis	Poor control over morphology, agglomeration of particles	[25]
Green synthesis	Use of natural extracts as reducing agents (like plants, biomass, and microbial metabolites)	Nontoxic, eco-friendly	Less purity, low yield	[26]

**Table 2:** Multi-cycle adsorption performance of MNMs for the removal of various types of water pollutants, along with desorbing agents employed for regeneration.

Type of MNM	Pollutant	Desorbing Agents	Regeneration Conditions (Contact Time/Temp/Washing/Drying)	No. of Cycles	Reusability (%)	Adsorption Mechanism (Interpretation)	**Ref.**
Fe_3_O_4_@PGP-NPs	Dye (AG 25)	0.1 M NaOH	88 min/70 °C/DI water washing	5	>62%	Electrostatic interactions and hydrogen bonding via surface functional groups (–OH, C=O/C–O, and Fe–O)	[39]
Fe_3_O_4_@DABA	Dye (CR, EY)	0.1 M NaOH	15 min/25 °C/DI water washing	5	CR: 90.16% EY: 95.52%	Electrostatic interactions (between protonated –NH_3_^+^ groups and anionic dye species), π–π stacking, and hydrogen bonding via amine and carboxyl functional groups on DABA-functionalized Fe_3_O_4_ surface	[40]
Fe_3_O_4_ nanoparticles	HM (Pb)	1 M HCl	45 min/25 °C/DI water washing (twice)	2	90.02%	Electrostatic attraction, surface complexation, and pH-dependent Pb (OH)_2_ precipitation	[10]
Fe_2_O_3_ nanoparticles	HM (Pb, Cd)	0.1 M HCl	60 min/25 °C	4	Pb: 52.1% Cd: 41.3%	Chemisorption via surface complexation and electrostatic interactions with Fe–OH active sites	[41]
IONPs	HM (Pb, Cd)	0.1 M HCl	60 min/25 °C/DI water washing	5	Pb: 88.35% Cd: 80.41%	Chemisorption via surface complexation and electron exchange with Fe-based active sites (monolayer adsorption)	[42]
O-Fe_3_O_4_	HM (Pb)	Distilled water	90 min/55 °C	5	Nearly 75%	Electrostatic interactions and chemisorption via surface functional groups (OH, –COOH) on Fe_3_O_4_ nanoparticles	[43]
Fe_3_O_4_@CP-NPs	Dye (RY145)	0.1 M NaOH	NaOH washing under optimized conditions	6	99.12	Electrostatic adsorption of azo dye molecules onto Fe_3_O_4_@CP-NPs surface	[44]
Fe_3_O_4_@SiO_2_-(TCT)_2_-(NH_2_)_2_ nanoparticles	HM (Cu, Cd)	0.1 mol L^−1^ HCl	Drying for 60 °C	8	>80%	Chemisorption via coordination/chelation with NH_2_-containing dendrimer and surface Si–OH groups, supported by electrostatic interactions	[45]
Fe_3_O_4_/SiO_2_-NH_2_	HM (Cu)	0.7 M HCl	120 min/25 °C/DI water washing	5	>90%	Chelation (coordination) via –NH_2_ functional groups on Fe_3_O_4_/SiO_2_ surface, supported by electrostatic interactions	[46]
Fe_2_O_3_ nanoparticles	Pharmaceutical (TC, DF)	Ethanol	24 h at 160 rpm/drying at 70 °C/DI water washing	5	>65%	Electrostatic interactions, hydrogen bonding, π–π interactions, and surface complexation between antibiotic molecules and Fe_2_O_3_ surface functional groups	[47]
Mn-doped SPIONPs	HM (Pb)	Distilled water	Rinsing three times	4	>75%	Chemisorption involving surface interactions between Pb^2+^ ions and active sites	[48]
Fe_3_O_4_@C	Dye (MB)	Methanol	Oven drying at 60 °C	5	50%	Electrostatic interactions and van der Waals (dispersion) forces between MB molecules and functionalized Fe_3_O_4_ surface	[49]
GO·Fe_3_O_4_	Pharmaceutical (CPT)	0.25 mol L^−1^ NaOH	1 h at 150 rpm/25 °C	5	Nearly 95%	Electrostatic interactions and hydrogen bonding between CPT molecules and oxygenated functional groups on GO–Fe_3_O_4_ nanocomposite surface	[50]
Fe-based MOF (MIL-100)	HM (As)	Ethanol	24 h/DI water washing	5	>97%	Inner-sphere monodentate Fe–O–As complexation at unsaturated Fe sites	[51]
Fe_3_O_4_/NiO	HM (Pb)	0.2 M HCl	30 min/25 °C/ultrapure water washing	5	Nearly 90%	Electrostatic attraction and surface complexation with deprotonated –OH/–COOH groups	[27]
USMNs	HM (Pb)	0.1 M HCl	Distilled water washing/oven drying at 60 °C	5	92%	Electrostatic interactions, surface complexation, and ion exchange with –OH groups	[52]
Fe_3_O_4_@SiO_2_-NH-nNG-SPTZ	HM (Hg)	0.3 mol L^−1^ HCl	25 °C/DI water washing	3	>90%	Heterogeneous multilayer adsorption via surface complexation/chelation with N/O/S groups and electrostatic attraction	[53]
F/α/C nanocomposite	HM (Pb)	0.1 M HCl	25 °C/DI water washing	4	97%	Electrostatic attraction + surface complexation (–OH, –COOH) + ion exchange + pore diffusion + pH-dependent precipitation of Pb(OH)_2_ on porous Fe–carbon composite	[54]
Alg/Clin/Fe_3_O_4_	Dye (CV)	0.1 M HCl	4 h/25 °C/DI water washing, oven drying	5	>80%	Electrostatic attraction (due to OH/COO^−^ groups), ion pairing with alginate carboxylates, and pore diffusion in porous Clin/Fe_3_O_4_-based composites	[55]
Fe_2_O_3_/AC nanocomposite	Dye (CR)	Ethanol	30 min/20 °C	3	>90%	Hydrogen bonding, electrostatic attraction (–COO^−^, C=O), and π–π stacking	[56]
NiO–Fe_3_O_4_ NPs	HM (Pb, Hg)	0.001 M HNO_3_	30 min/DI water washing (three times), drying overnight at 100 °C	4	>90%	Electrostatic attraction on deprotonated Fe–OH/Ni–OH sites and chemisorption (inner-sphere complexation)	[57]
Fe_3_O_4_@DAL	Pharmaceutical (PEF, LOM, ENR, DIF)	Methanol/acetic acid (9:1, *v*/*v*)	Desorbing agent used twice with shaking	5	PEF: 82% LOM: 84% ENR: 86% DIF: 91%	Electrostatic attraction between –COO^−^/–O^−^ and =N^+^ groups and hydrophobic interactions between FQs and aromatic DAL domains	[29]
3D/Fe/GO/ME/DCS	Pharmaceutical (LEV)	Methanol	1 min of sonication	7	>84%	Monolayer physisorption on a homogeneous surface governed by weak interactions (van der Waals/hydrogen bonding)	[8]

**Table 4:** Comparative adsorption performance and economic evaluation of MNMs in the removal of various water pollutants.

Type of MNM	Pollutant Removed	Adsorption Capacity (mg g^−1^)	Reported Adsorbent Cost (USD kg^−1^)	Adsorption Cost (USD g^−1^)	Ref.
MagA	HM (Pb)	158.7	6.7	0.0422	[75]
MgFe_2_O_4_-PANI-NC	Dye (MR)	98.04	100.60	1.03	[76]
Fe_3_O_4_-RAC	Dye (MB)	32.25	22.29	0.691	[77]
POM-MBC	Pharmaceutical (MNZ)	78.45	80.03	1.02	[78]
MBC-IO	HM (Pb)	504	1.912	0.00379	[79]
PVA-MH	HM (Cd)	13.8	8.38	0.607	[80]
Fe_3_O_4_@SiO_2_/ALP-p	Pharmaceutical (BPA)	485.1	11.12	0.0231	[81]
AF-Fe_3_O_4_	HM (Cr)	212.1	16.19	0.0763	[82]

## Data Availability

No new data were created or analyzed in this study.
